# Dimension-Dependent Bandgap Narrowing and Metallization in Lead-Free Halide Perovskite Cs_3_Bi_2_X_9_ (X = I, Br, and Cl) under High Pressure

**DOI:** 10.3390/nano11102712

**Published:** 2021-10-14

**Authors:** Guangbiao Xiang, Yanwen Wu, Man Zhang, Chen Cheng, Jiancai Leng, Hong Ma

**Affiliations:** 1 Shandong Provincial Key Laboratory of Optics and Photonic Device, Collaborative Innovation Center of Light Manipulations and Applications, School of Physics and Electronics, Shandong Normal University, Jinan 250014, China; m17753643157@163.com (G.X.); yanwenwu1209@163.com (Y.W.); zhangman010501@163.com (M.Z.); 2 School of Electronic and Information Engineering (Department of Physics), Qilu University of Technology (Shandong Academy of Sciences), Jinan 250353, China

**Keywords:** cesium bismuth iodide perovskite, first-principles study, high pressure, bandgap narrowing, metallization

## Abstract

Low-toxicity, air-stable cesium bismuth iodide Cs_3_Bi_2_X_9_ (X = I, Br, and Cl) perovskites are gaining substantial attention owing to their excellent potential in photoelectric and photovoltaic applications. In this work, the lattice constants, band structures, density of states, and optical properties of the Cs_3_Bi_2_X_9_ under high pressure perovskites are theoretically studied using the density functional theory. The calculated results show that the changes in the bandgap of the zero-dimensional Cs_3_Bi_2_I_9_, one-dimensional Cs_3_Bi_2_Cl_9_, and two-dimensional Cs_3_Bi_2_Br_9_ perovskites are 3.05, 1.95, and 2.39 eV under a pressure change from 0 to 40 GPa, respectively. Furthermore, it was found that the optimal bandgaps of the Shockley–Queisser theory for the Cs_3_Bi_2_I_9_, Cs_3_Bi_2_Br_9_, and Cs_3_Bi_2_Cl_9_ perovskites can be reached at 2–3, 21–26, and 25–29 GPa, respectively. The Cs_3_Bi_2_I_9_ perovskite was found to transform from a semiconductor into a metal at a pressure of 17.3 GPa. The lattice constants, unit-cell volume, and bandgaps of the Cs_3_Bi_2_X_9_ perovskites exhibit a strong dependence on dimension. Additionally, the Cs_3_Bi_2_X_9_ perovskites have large absorption coefficients in the visible region, and their absorption coefficients undergo a redshift with increasing pressure. The theoretical calculation results obtained in this work strengthen the fundamental understanding of the structures and bandgaps of Cs_3_Bi_2_X_9_ perovskites at high pressures, providing a theoretical support for the design of materials under high pressure.

## 1. Introduction

Organic–inorganic hybrid perovskites have attracted much attention from the researcher community because of their remarkable photoelectric properties, including high absorption coefficients in the visible light region, tunable bandgaps, high quantum yields, high carrier mobilities, and low effective carrier quality [1,2,3,4,5,6]. In addition, their processing is economical and utilizes simple solution treatments [7,8]. The power conversion efficiency (PCE) of perovskite solar cells (PSCs) has increased dramatically from 3.8% in 2009 to 25.5% [9,10]. Lead hybrid perovskites have the outstanding photovoltaic properties and a high PCE. However, the high toxicity of lead is a major challenge for the large-scale fabrication and commercialization of lead-based PSCs. Recently, nontoxic, all-inorganic, lead-free Bi-based perovskites have attracted significant attention owing to their stability, high photoluminescence, high quantum yield, and tunable bandgaps [11,12,13,14,15,16,17]. They have been widely used in applications, such as memory devices, photodetectors, solar cells, and X-ray detectors [18,19,20,21,22,23,24,25,26]. However, many studies have shown that there are some challenges to overcome for the use of Cs_3_Bi_2_X_9_ (X = I, Br, and Cl) perovskites in photovoltaic devices. The large bandgap (>2 eV) is the most important factor causing a low PCE, which limits their absorption efficiency and carrier transport performance. According to the Shockley–Queisser theory, a semiconductor with a bandgap in the range of 1.3–1.5 eV is an ideal material for solar cells. Although the PCE of Bi-based PSCs has improved slightly with the improvement of the thin-film technology, it still lags far behind that of lead-based perovskites. The traditional chemical modification cannot overcome this inherent limit. Therefore, tuning the bandgap with the aim of improving the photovoltaic performance of the Cs_3_Bi_2_X_9_ perovskites has become a key challenge.

High pressure (HP), which is a non-polluting tuning method, is widely used to modulate the physical and chemical properties of materials without changing their chemical composition [27,28]. In recent years, the properties of halide perovskites have been widely studied under HP; the properties studied include piezochromism, bandgap engineering, structural phase transitions and optical properties, [29,30,31,32]. In addition, various novel physical phenomena have been observed under HP conditions. For example, the organic–inorganic hybrid perovskite nanocrystals present the comminution and recrystallization under HP and exhibit higher photoluminescence quantum yield and a shorter carrier lifetime [29]. A reversible amorphization has been observed for the CH_3_NH_3_PbBr_3_ perovskite under a pressure of approximately 2 GPa; during this transition, the resistance increased by five orders of magnitude, and the material still retained its response to the visible light and semiconductor characteristics up to a pressure of 25 GPa [33]. Notably, pressure-induced structural changes and optical properties are reversible upon decompression, and a semiconductor–metal transition can be observed at 28 GPa [34]. It is notable that there are few studies on Cs_3_Bi_2_X_9_ perovskite systems of different dimensions at HP [34,35], and no reports exist on the one-dimensional halide perovskite Cs_3_Bi_2_Cl_9_.

In this work, we calculated the lattice constants, band structures, density of states (DOS), and optical properties of the one-dimensional perovskite Cs_3_Bi_2_Cl_9_ via the density functional theory (DFT) under HP for the first time and compared with the zero-dimensional perovskite Cs_3_Bi_2_I_9_ and the two-dimensional perovskite Cs_3_Bi_2_Br_9_. We discussed the relationship between the bandgap of the Cs_3_Bi_2_X_9_ perovskites and the HP and focused on the optimal bandgap of the Shockley–Queisser theory of the Cs_3_Bi_2_X_9_ perovskites. The Cs_3_Bi_2_I_9_ perovskite completed the transition from semiconductor to metal at 17.3 GPa, this finding indicated that HP is an effective means to induce the semiconductor–metal transition. Moreover, it was found that the lattice constants and bandgaps of the Cs_3_Bi_2_X_9_ perovskites are dependent upon dimension, that is, the changes in the lattice constants and bandgap gradually decrease as the dimension increases from zero to two under the same pressure. Our calculated results obtained in this work strengthen the basic understanding of different structures of the Cs_3_Bi_2_X_9_ (X = I, Br and Cl), providing theoretical guidance for the structure and bandgap regulation of Bi-based perovskites under HP.

## 2. Computational Model and Method

The DFT was performed in the Vienna *Ab-initio* Simulation Package (VASP) using the projected augmented wave (PAW) framework [36,37]. DFT is derived from the Schrodinger equation under the Born–Oppenheimer approximation, described by the Hohenberg–Kokn theorem and the Kohn–Sham equation. The pseudopotential is a hypothetical potential energy function used in place of the inner electron wave function to reduce the computation. The electron exchange–correction function was obtained via the generalized gradient approximation (GGA) parameterized using the Perdew–Burke–Ernzerhof (PBE) formalism [38]. The cut-off energy of the plane wave was set to 500 eV [34]. The convergence criteria for the energy and force were set to 10^−5^ eV and 0.01 eV/Å, respectively. The Brillouin zone integration were sampled with 4 × 4 × 4, 4 × 4 × 4, and 4 × 4 × 2 Gamma-pack k-point meshes during the structure optimization of Cs_3_Bi_2_I_9_, Cs_3_Bi_2_Br_9_, and Cs_3_Bi_2_Cl_9_, respectively. The entire optimization of the structures was completely relaxed [39]. The valence electronic configurations of the Cs, Bi, I, Br, and Cl atoms are 5s^2^5p^6^6s^1^, 5d^10^6s^2^6p^3^, 5s^2^5p^5^, 4s^2^4p^5^, and 3s^2^3p^5^, respectively. The Γ Brillouin zone center has a highly symmetric path, with coordinates Γ (0, 0, 0) to the M (0.5, 0, 0), K (0.333, 0.333, 0), Γ (0, 0, 0), A (0, 0, 0.5), L (0.5, 0, 0.5), and H (0.333, 0.333, 0.5). The spin–orbit coupling is not considered due to the high computational cost. Previous studies have shown that higher levels of calculation (including the spin–orbit coupling, the GW method, or hybrid functionals) obtain more accurate bandgaps; However, they induce little change in the band structure of the heavy-metal halide perovskites [40].

## 3. Results and Discussion

**Figure 1:**
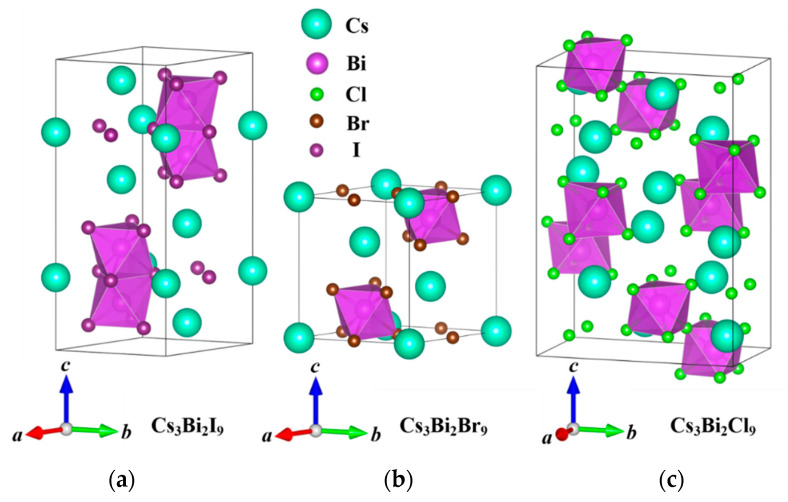
Crystal structures of the zero-dimensional perovskite Cs_3_Bi_2_I_9_ (**a**), two-dimensional perovskite Cs_3_Bi_2_Br_9_ (**b**), and one-dimensional perovskite Cs_3_Bi_2_Cl_9_ (**c**).

**Figure 2:**
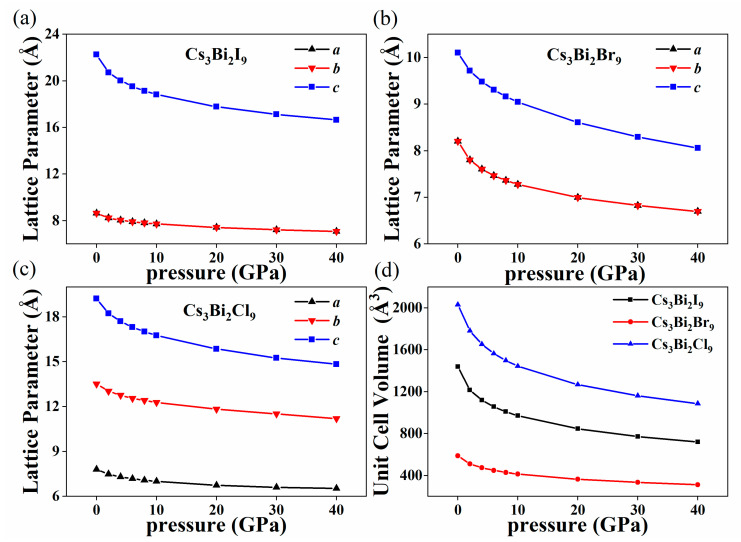
Changes in the calculated lattice parameter as a function of pressure from 0 GPa to 40 GPa for the Cs_3_Bi_2_I_9_ perovskite (**a**), Cs_3_Bi_2_Br_9_ perovskite (**b**), and Cs_3_Bi_2_Cl_9_ perovskite (**c**). Changes in the unit cell volume of the Cs_3_Bi_2_X_9_ perovskites under the pressures from 0 GPa to 40 GPa (**d**).

The structures of the three halogenated perovskite crystals Cs_3_Bi_2_X_9_ (X = I, Br, and Cl) are shown in Figure 1. The one-dimensional Cs_3_Bi_2_Cl_9_ perovskite has an orthogonal-crystal structure; the two-dimensional Cs_3_Bi_2_Br_9_ perovskite and the zero-dimensional Cs_3_Bi_2_I_9_ perovskite have hexagonal cells [41,42]. The numbers of atoms in the Cs_3_Bi_2_I_9_, Cs_3_Bi_2_Br_9_, and Cs_3_Bi_2_Cl_9_ perovskites in the primitive cell are 28, 14, and 56, respectively. The Bi atom is located at the center of the octahedron in the Cs_3_Bi_2_X_9_ perovskites and is surrounded by six halogen atoms. Unlike in lead-based perovskites, in the Cs_3_Bi_2_Br_9_ and Cs_3_Bi_2_Cl_9_ perovskites, two octahedrons share one X atom (X = Br or Cl), whereas two octahedrons share three X atoms in the Cs_3_Bi_2_I_9_ perovskite. X atoms with different sizes and the Bi atoms form a novel double-perovskite structure. Figure 2 shows the changes in the lattice parameters and volume of the Cs_3_Bi_2_X_9_ perovskites under HP. In the primary cell of the Cs_3_Bi_2_I_9_ and Cs_3_Bi_2_Br_9_ perovskites, the lattice constants a and b are equal. The lattice constant and volume of Cs_3_Bi_2_X_9_ clearly decrease with an increase in pressure, and the slope also decreases gradually. As is well established, pressure induces a reduction in the lattice constant. From a microscopic point of view, the pressure shrinks the distance between two atoms. The strong Coulomb force makes it increasingly difficult to further compress the material as the pressure increases.

Based on the semiconductor theory, analyzing the band structure and the DOS very closely, the Fermi level is important for establishing the possible applications of a material in the photoelectric and photovoltaic fields. We therefore calculated the band structure near the Fermi level (from −5 eV to +5 eV) under different pressures. The calculated bandgaps at a pressure of 0 GPa of the Cs_3_Bi_2_I_9_, Cs_3_Bi_2_Br_9_, and Cs_3_Bi_2_Cl_9_ are 2.38, 2.60, and 3.08 eV, respectively. The scissor value approach was used to establish the band structure of the Cs_3_Bi_2_X_9_ perovskites in order to obtain the accurate value for the Cs_3_Bi_2_X_9_ perovskites when they reached the optimal bandgap of the Shockley–Queisser theory and the transition from semiconductor to metal; this methodology overcame the limitations of the GGA–PBE calculation method and has already been applied in the study of the photoelectric properties of the CsSnCl_3_ perovskite at HPs [40]. Many experimental studies have been conducted regarding the bandgaps of the Cs_3_Bi_2_I_9_, Cs_3_Bi_2_Br_9_, and Cs_3_Bi_2_Cl_9_ perovskites. Among these, Daniel R. Gamelin et al. synthesized Cs_3_Bi_2_X_9_ perovskites nanocrystals via the thermal injection method, and the bandgaps of the Cs_3_Bi_2_I_9_, Cs_3_Bi_2_Br_9_, and Cs_3_Bi_2_Cl_9_ perovskites were measured as 2.07, 2.76, and 3.26 eV, respectively [43]. Therefore, the scissors values of −0.31eV, 0.16eV, and 0.18eV were used for the Cs_3_Bi_2_I_9_, Cs_3_Bi_2_Br_9_, and Cs_3_Bi_2_Cl_9_ perovskites, respectively.

**Figure 3:**
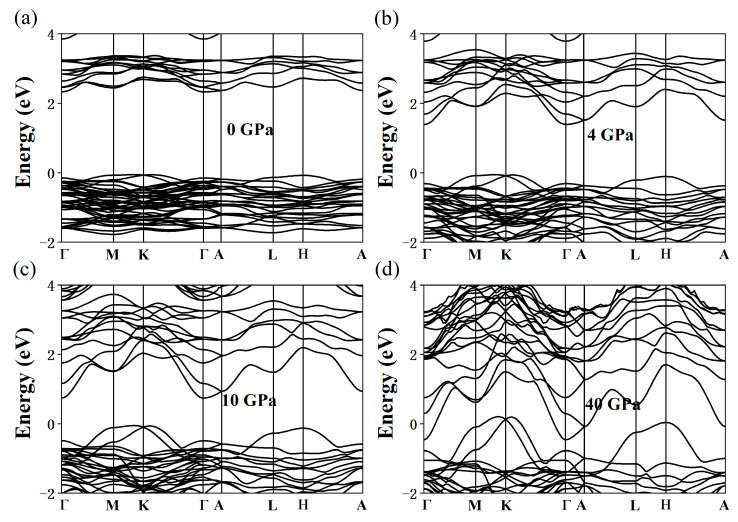
Calculated band structure of the Cs_3_Bi_2_I_9_ perovskite under the pressures of 0 (**a**), 4 (**b**), 10 (**c**), and 40 GPa (**d**).

**Figure 4:**
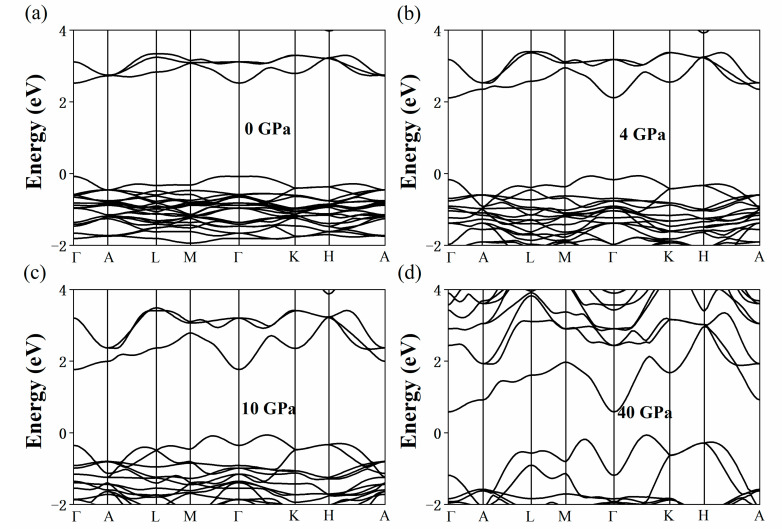
Calculated band structure of the Cs_3_Bi_2_Br_9_ perovskite under the pressures of 0 (**a**), 4 (**b**), 10 (**c**), and 40 GPa (**d**).

**Figure 5:**
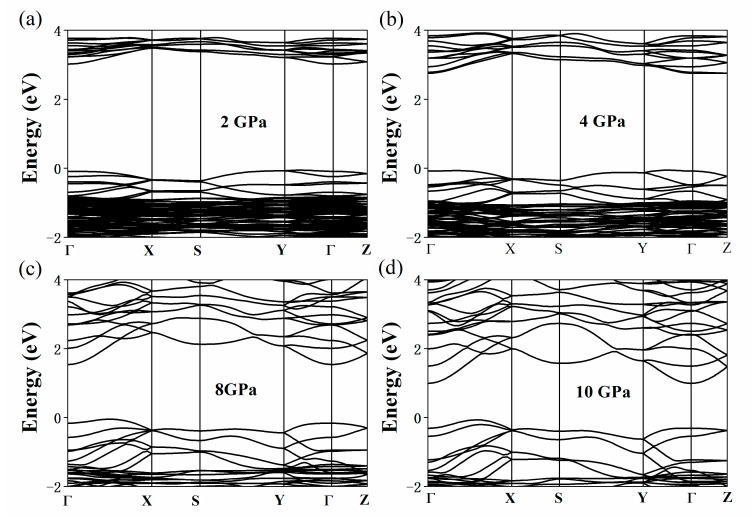
Calculated band structure of the Cs_3_Bi_2_Cl_9_ perovskite under the pressures of 0 (**a**), 4 (**b**), 10 (**c**), and 40 GPa (**d**).

Figure 3 shows the change in the band structure of the Cs_3_Bi_2_I_9_ perovskite under the pressures of 0 (a), 4 (b), 10 (c), and 40 GPa (d). Without external pressure, the conduction band minimum (CBM) and the valence band maximum (VBM) of Cs_3_Bi_2_I_9_ perovskite are located at the Γ- and M-points, respectively. We found the Cs_3_Bi_2_I_9_ perovskite to be an indirect bandgap material, which is consistent with the existing literature [44]. The semiconductor with the optimized band gap energy of 1.34 eV is critical to achieve the efficiency limit of 33.7% based on the Shockley–Queisser theory [34]. In Figure 3, it can be seen that the bandgap of the Cs_3_Bi_2_I_9_ perovskite decreases sharply with the increase in pressure and reaches its optimal bandgap value given by the Shockley–Queisser theory at 2–3 GPa. In addition, with the high pressure further increasing, the CBM continues to decrease and the VBM moves from the M-point to near the K-point. The Cs_3_Bi_2_I_9_ perovskite completed the transition from semiconductor to metal at 17.3 GPa, and this finding indicated that HP is an effective means to induce the semiconductor–metal transition. The band structure of the Cs_3_Bi_2_Br_9_ perovskite under the pressures of 0 (a), 4 (b), 10 (c), and 40 GPa (d) is presented in Figure 4. As can be seen from Figure 4, when the pressure is 0 GPa (a), the calculated band structure of the Cs_3_Bi_2_Br_9_ perovskite is consistent with the results reported by Brent C. Melot et al. [45] They found a low-lying 2.52 eV indirect transition as well as a slightly larger direct gap of 2.64 eV, which are essentially in agreement with our calculated results. This structural characteristic becomes increasingly evident with the increase in pressure. The Cs_3_Bi_2_Br_9_ perovskite reaches the optimal bandgap given by the Shockley–Queisser theory at 21–26 GPa, which means that the electron transitions from the valence band to conduction band become easier. Figure 5a–d shows the change of the band structure of Cs_3_Bi_2_Cl_9_ perovskite with HP. The Cs_3_Bi_2_Cl_9_ perovskite reaches the optimal bandgap in the range of 25–29 GPa; it is an indirect bandgap material. The CBM is at the Γ- point, and the VBM moves from the Γ-point to the Y-point, which is consistent with the results reported previously [41]. However, it was found that the Cs_3_Bi_2_Br_9_ and Cs_3_Bi_2_Cl_9_ perovskites did not metallize under HP despite the pressure reaching 40 GPa in both cases, which may be related to their unique structure. The bandgap changes in the Cs_3_Bi_2_X_9_ perovskites under a range of HP (0, 2, 4, 6, 8, 10, 20, 30, and 40 GPa) are shown in Supplementary Figures S1–S3.

**Figure 6:**
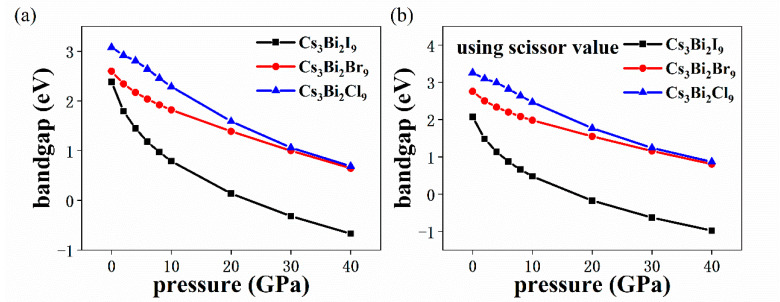
Calculated bandgap of the Cs_3_Bi_2_X_9_ perovskites as a function of pressure (from 0 GPa to 40 GPa) (**a**). Changes in the bandgap of the Cs_3_Bi_2_X_9_ perovskite after using the scissor values under HP (**b**). The black, red, and blue lines represent the Cs_3_Bi_2_I_9_, Cs_3_Bi_2_Br_9_, and Cs_3_Bi_2_Cl_9_ perovskites, respectively.

Figure 6 shows the changes in the bandgaps of the Cs_3_Bi_2_I_9_, Cs_3_Bi_2_Br_9_, and Cs_3_Bi_2_Cl_9_ perovskites under HP. It can be seen that the bandgap of the Cs_3_Bi_2_X_9_ perovskites decreases with the increase in pressure (Figure 6a); the bandgap of the Cs_3_Bi_2_I_9_ perovskite takes a negative value, which is a typical indicator of metallic behavior. Figure 6b shows the change in the bandgap of the Cs_3_Bi_2_X_9_ perovskites after using the scissor values under HP. When the pressure is varied from 0 to 40 GPa, the bandgap differences of the Cs_3_Bi_2_I_9_, Cs_3_Bi_2_Br_9_, and Cs_3_Bi_2_Cl_9_ perovskites are 3.05, 1.95, and 2.39 eV, respectively. It was found that with the increase in dimension, the bandgap differences of the Cs_3_Bi_2_I_9_, Cs_3_Bi_2_Br_9_, and Cs_3_Bi_2_Cl_9_ perovskites decrease in turn, which indicates that the bandgap of the Cs_3_Bi_2_X_9_ perovskites depends on dimension.

**Figure 7:**
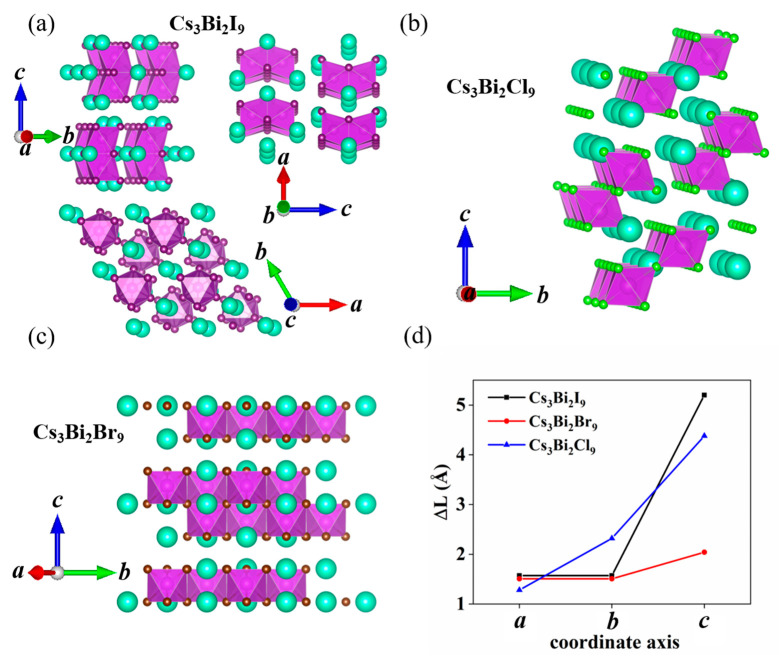
Crystal structure of the zero-dimensional Cs_3_Bi_2_I_9_ (**a**), one-dimensional Cs_3_Bi_2_Cl_9_ (**b**), and two-dimensional Cs_3_Bi_2_Br_9_ (**c**). Difference in the lattice constants of the Cs_3_Bi_2_X_9_ perovskites for the pressures between 0 and 40 GPa along the a, b, and c axes (**d**). The black, red, and blue lines represent Cs_3_Bi_2_I_9_, Cs_3_Bi_2_Br_9_, and Cs_3_Bi_2_Cl_9_, respectively.

To explain the dimension-dependent bandgap of the Cs_3_Bi_2_X_9_, we investigate the construction of these perovskites. Based upon the primitive cell of Cs_3_Bi_2_I_9_, Cs_3_Bi_2_Br_9_ and the Cs_3_Bi_2_Cl_9_ perovskites, the 2 × 2 × 1, 2 × 2 × 2, and 3 × 1 × 1 supercells are shown in Figure 7a–c, respectively. In Figure 7d, ΔL represents the difference in the lattice constants of the Cs_3_Bi_2_X_9_ perovskites between 0 and 40 GPa along the a, b, and c coordinate axes. In general, for centrosymmetric perovskites, the ΔL values along the *a*- (ΔL_a_), *b*- (ΔL_b_), and *c*- (ΔL_c_) axes are equal under HP. However, we found that ΔL_a_, ΔL_b_, and ΔL_c_ for the double perovskites were not equal; in other words, ΔL is anisotropic along the *a*-, *b*-, and *c*-axes. The ΔL_a_, ΔL_b_, and ΔL_c_ values for the Cs_3_Bi_2_I_9_ perovskite (zero-dimensional) are 1.57, 1.57, and 5.2. It can be seen that ΔL_c_ is much larger than both ΔL_a_ and ΔL_b_. The reason for this difference is that the Bi_2_I_9_^3−^ frame of the double-perovskite is continuous along the *a*- and *b*-axes but not along the c-axis. Along the c-axis, only the Cs^+^ atoms are above or below the Bi_2_I_9_^3−^ frame, which indicates that the change in the lattice constant along the c-axis is bigger than that observed along the *a*- and *b*-axes when the zero-dimensional Cs_3_Bi_2_I_9_ perovskite is placed under HP.

The previous analysis shows that if the double-perovskite frame expands regularly in one direction, the ΔL in this direction will be smaller than in the other directions under HP. This indicates that the lattice constants of the Cs_3_Bi_2_X_9_ perovskites are dependent on dimension. In general, the lattice constant and the bandgap decrease as the pressure increases [34]. It has also been found that the changes in the bandgap of the zero-dimensional Cs_3_Bi_2_I_9_, one-dimensional Cs_3_Bi_2_Cl_9_, and two-dimensional Cs_3_Bi_2_Br_9_ perovskites between 40 and 0 GPa are 3.05, 2.39, and 1.95 eV, respectively. These results illustrate that the bandgaps of the Cs_3_Bi_2_X_9_ perovskites are also dependent upon dimension; that is, the changes in the bandgap decrease gradually as the dimension increased from zero to two under the same pressure.

**Figure 8:**
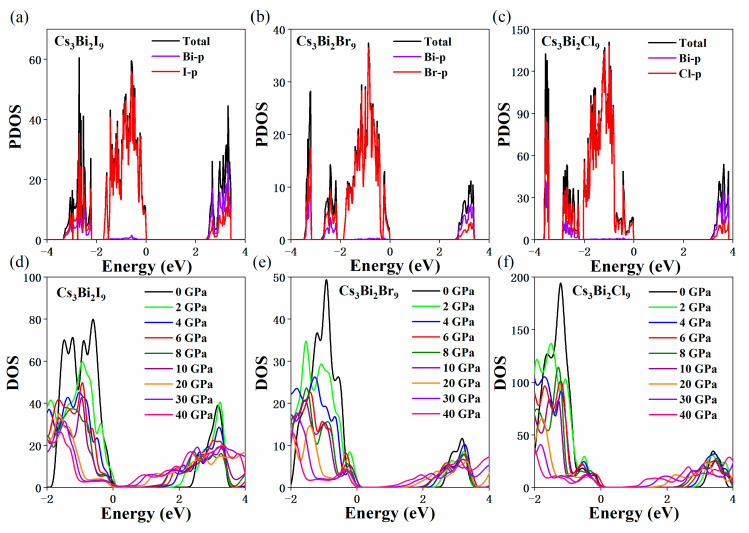
Partial state of density (PDOS) of Cs_3_Bi_2_I_9_ (**a**), Cs_3_Bi_2_Br_9_ (**b**), and Cs_3_Bi_2_Cl_9_ (**c**). the VBM of the Cs_3_Bi_2_X_9_ perovskites (X = I, Br, and Cl) is dominated by the p-X states, and the CBM is dominated by the p-Bi and p-X states. Calculated density of states (DOS) of the Cs_3_Bi_2_I_9_ (**d**), Cs_3_Bi_2_Br_9_ (**e**), and Cs_3_Bi_2_Cl_9_ (**f**) under the pressures from 0 to 40 GPa.

The partial density of states (PDOS) of the zero-dimensional Cs_3_Bi_2_I_9_ (a), one-dimensional Cs_3_Bi_2_Cl_9_ (b), and two-dimensional Cs_3_Bi_2_Br_9_ (c) perovskites were shown in Figure 8. It can be seen that the VBM of the Cs_3_Bi_2_X_9_ perovskites is dominated by p-X states, whereas the CBM is dominated by the p-Bi and p-X states (see Figure 8a–c). The changes in the DOS under HP are shown in Figure 8d–f. It is clear that many valence bands in the Cs_3_Bi_2_X_9_ perovskites move to a deep level, and the conduction bands approach to the FE with an increase in pressure for Cs_3_Bi_2_X_9_ perovskites; the shift of the conduction bands under the HP will induce the changes in the bandgap. In contrast to those of the Cs_3_Bi_2_Br_9_ and Cs_3_Bi_2_Cl_9_ perovskites, the forbidden band width of the Cs_3_Bi_2_I_9_ gradually decreases and subsequently disappears, which indicates that the Cs_3_Bi_2_I_9_ perovskite is no longer a semiconductor; it becomes a metal at 17.3 GPa. This conclusion is agreement with the calculated results for the band structure.

A large absorption coefficient is of great significance in photoelectric and photovoltaic applications. Such a property improves the PCE of solar cells and the luminous efficiency. The absorption coefficient is usually described by the dielectric function according to the following expression [46]:

$$\alpha =2\omega {\left[\frac{{\left(\varepsilon_{1}^{2}\left(\omega \right)+\varepsilon_{2}^{2}\left(\omega \right)\right)}^{1/2}-\varepsilon_{1}\left(\omega \right)}{2}\right]}^{1/2}$$

where the ω is the frequency of light, and $\varepsilon_{1}^{}$ and $\varepsilon_{2}^{}$ are the real and imaginary parts of the dielectric function, respectively. The calculated $\varepsilon_{1}^{}$ and $\varepsilon_{2}^{}$ values of the Cs_3_Bi_2_X_9_ perovskites are presented in Supplementary Figures S4–S6 along the *a*-, *b*-, and *c*-axes. The static dielectric function of the Cs_3_Bi_2_X_9_ perovskites increases gradually with increasing pressure from 0 to 40 GPa. The $\varepsilon_{2}^{}$ value is closely related to the optical absorption and usually used to describe the absorption behavior of materials.

**Figure 9:**
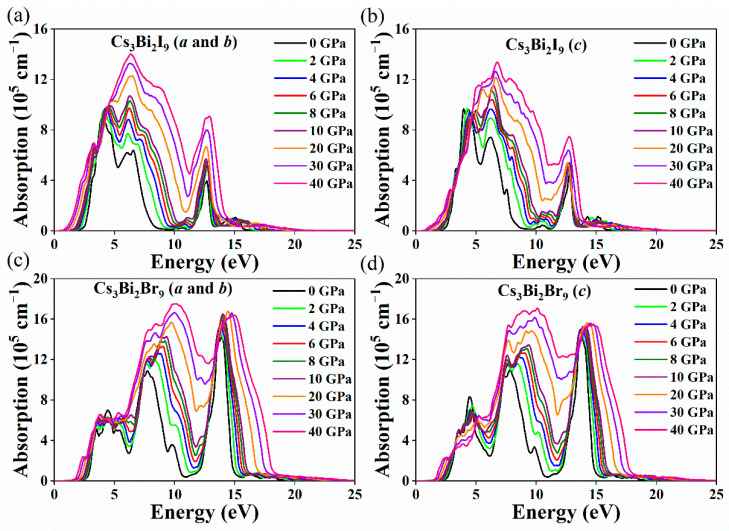
Absorption coefficients of the Cs_3_Bi_2_I_9_ along the *a*- and *b*-axes (**a**), Cs_3_Bi_2_I_9_ along *c*-axis (**b**), Cs_3_Bi_2_Br_9_ along the *a*- and *b*-axes (**c**), and Cs_3_Bi_2_Br_9_ along the *c*-axis (**d**) as a function of the pressure (from 0 to 40 GPa).

**Figure 10:**
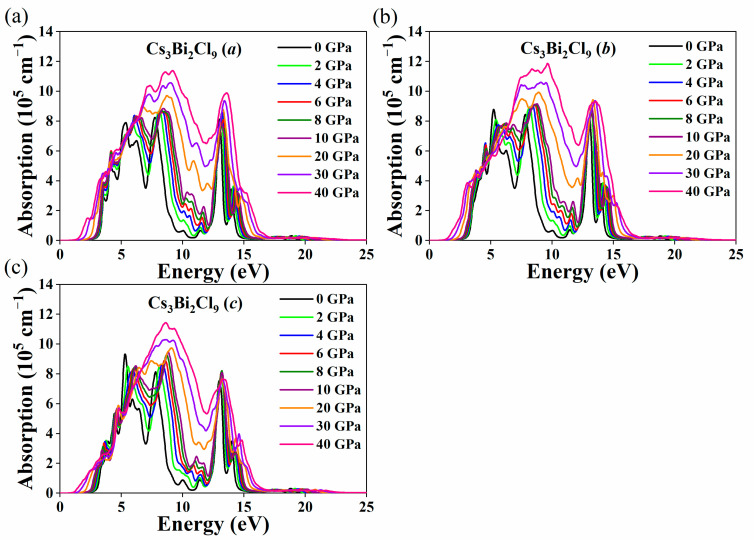
Absorption coefficients of Cs_3_Bi_2_Cl_9_ along the *a*-axis (**a**), along the *b*-axis (**b**), and along the *c*-axis (**c**) as a function of the pressure (from 0 to 40 GPa).

The calculated absorption coefficients of the Cs_3_Bi_2_X_9_ perovskites are shown in Figure 9 and Figure 10 along the *a*-, *b*-, and *c*-axes. It was found that the Cs_3_Bi_2_I_9_ perovskite has the same absorption coefficient along the a- and b-axes with the increase in pressure, as is the case for the Cs_3_Bi_2_Br_9_ perovskite. The absorption coefficients of the Cs_3_Bi_2_Cl_9_ perovskite are unequable along the *a*-, *b*-, and *c*-axes under HP. The ΔL value of the Cs_3_Bi_2_X_9_ perovskites in Figure 7d is consistent with these behaviors, which suggests that the changes in the structure of the Cs_3_Bi_2_X_9_ perovskites under HP will affect the optical properties considerably. The Cs_3_Bi_2_X_9_ perovskites exhibit a redshift with the increase in pressure, which indicates that the Cs_3_Bi_2_X_9_ can absorb the low-energy photons. Moreover, the absorption coefficients of the Cs_3_Bi_2_X_9_ perovskites increase gradually in the ultraviolet region as the pressure increases from 0 to 40 GPa. It was also found that the Cs_3_Bi_2_X_9_ perovskites have a large absorption coefficient in the visible region (on the order of 10^5^ cm^−1^). Therefore, the Cs_3_Bi_2_X_9_ perovskites are an attractive candidate in applications of photoelectric and photovoltaic devices.

## 4. Conclusions

In summary, we investigated the lattice constants, band structure, DOS, and optical absorption of the cesium bismuth iodide Cs_3_Bi_2_X_9_ (X = I, Br and Cl) perovskites under HP by using the DFT. It was found that the optimal bandgap of the Shockley–Queisser theory for the Cs_3_Bi_2_I_9_, Cs_3_Bi_2_Br_9_, and Cs_3_Bi_2_Cl_9_ perovskites can be obtained at 2–3 GPa, 21–26 GPa, and 25–29 GPa, respectively. The changes in the bandgap of Cs_3_Bi_2_I_9_, Cs_3_Bi_2_Br_9_, and Cs_3_Bi_2_Cl_9_ perovskites are 3.05, 1.95, and 2.39 eV under a pressure of 40 GPa, respectively. The Cs_3_Bi_2_I_9_ perovskite was found to transform from a semiconductor into a metal at 17.3 GPa. Furthermore, the dimension-dependent lattice constants, unit-cell volumes, and bandgaps of the Cs_3_Bi_2_X_9_ perovskites were studied. Our calculations show that HP is an effective way to tune the photovoltaic and optoelectronic properties of the Cs_3_Bi_2_X_9_ perovskites by modifying the crystal structure, which provides a promising method for material design and applications.

## Data Availability

Data are contained within the article.

## Supplementary Materials

The following are available online at https://www.mdpi.com/article/10.3390/nano11102712/s1. Table S1: Summarized bandgap values of Cs_3_Bi_2_X_9_ perovskites under high pressure; Figure S1: Calculated band structures of the Cs_3_Bi_2_I_9_ perovskite under the different pressures (**a**–**i**); Figure S2: Calculated band structures of the Cs_3_Bi_2_Br_9_ perovskite under the different pressures (**a**–**i**); Figure S3: Calculated band structures of the Cs_3_Bi_2_Cl_9_ perovskite under the different pressures (**a**–**i**); Figure S4: Real part of the dielectric function of the Cs_3_Bi_2_I_9_ perovskite along the *a*- and *b*-axes (**a**), and the *c*-axis (**b**) as a function of pressure (from 0 to 40 GPa). Imaginary part of the dielectric function of the Cs_3_Bi_2_I_9_ perovskite along the *a*- and *b*-axes (**c**), and along the *c*-axis) (**d**) as a function of pressure (from 0 to 40 GPa); Figure S5: Real part of the dielectric function of the Cs_3_Bi_2_Br_9_ perovskite along the *a*- and *b*-axes (**a**), and the *c*-axis (**b**) as a function of pressure (from 0 to 40 GPa). Imaginary part of the dielectric function of the Cs_3_Bi_2_Br_9_ perovskite along the *a*- and *b*-axes (**c**), and along the *c*-axis) (**d**) as a function of pressure (from 0 to 40 GPa); Figure S6: Real part of the dielectric function of the Cs_3_Bi_2_Cl_9_ perovskite along the *a*-axis (**a**), along the *b*-axis (**b**), and along the *c*-axis (**c**) as a function of pressure (from 0 to 40 GPa). Imaginary part of the dielectric function of the Cs_3_Bi_2_Cl_9_ perovskite along the *a*-axis (**d**), along the *b*-axis (**e**), and along the *c*-axis (**f**) as a function of pressure (from 0 to 40 GPa).
