# Supplementary material for: Dimension-Dependent Bandgap Narrowing and Metallization in Lead-Free Halide Perovskite Cs3Bi2X9 (X = I, Br, and Cl) under High Pressure
Source: Nanomaterials (Basel). 2021 Oct 14;11(10):2712. doi: 10.3390/nano11102712 (PMC8539073; doi:10.3390/nano11102712)
Supplement: Supplementary file 1 [file nanomaterials-11-02712-s001.zip › nanomaterials-1416213-supplementary.pdf]

*Supplementary Information for*

# Dimension-Dependent Bandgap Narrowing and Metallization in Lead-Free Halide Perovskite $Cs_3Bi_2X_9$ (X = I, Br, and Cl) under High Pressure

**Guangbiao Xiang [1], Yanwen Wu [1], Man Zhang [1], Chen Cheng [1,*], Jiancai Leng [2,*] and Hong Ma [1,*]**

1 Shandong Provincial Key Laboratory of Optics and Photonic Device, Collaborative Innovation Center of Light Manipulations and Applications, School of Physics and Electronics, Shandong Normal University, Jinan 250014, China; m17753643157@163.com (G.X.); yanwenwu1209@163.com (Y.W.); zhangman010501@163.com (M.Z.)

2 School of Electronic and Information Engineering (Department of Physics), Qilu University of Technology (Shandong Academy of Sciences), Jinan 250353, China

* Correspondence: drccheng@sdnu.edu.cn (C.C.); jiancaileng@qlu.edu.cn (J.L.); mahong@sdnu.edu.cn (H.M.)

**Table S1.** Summarizing bandgap values of $Cs_3Bi_2X_9$ perovskites under high pressure in literature and compare with values in this work.

| Bandgap (eV) / High Press (GPa) | $Cs_3Bi_2I_9$ [1] | $Cs_3Bi_2Br_9$ [2] | $Cs_3Bi_2I_9$ (this work) | $Cs_3Bi_2Br_9$ (this work) | $Cs_3Bi_2Cl_9$ (this work) |
|---|---|---|---|---|---|
| 0 | 1.88 | 2.66 | 2.07 | 2.76 | 3.26 |
| 2 | 1.63 | 2.59 | 1.48 | 2.50 | 3.10 |
| 4 | 1.39 | 2.47 | 1.14 | 2.33 | 2.99 |
| 6 | 1.21 | 2.44 | 0.87 | 2.20 | 2.82 |
| 8 | 1.01 | 2.34 | 0.66 | 2.08 | 2.64 |
| 10 | 0.75 | 2.12 | 0.48 | 1.98 | 2.47 |
| 20 | 0.12 | 1.96 | -0.17 | 1.55 | 1.77 |
| 30 | | | -0.63 | 1.16 | 1.24 |
| 40 | | | -0.98 | 0.81 | 0.87 |

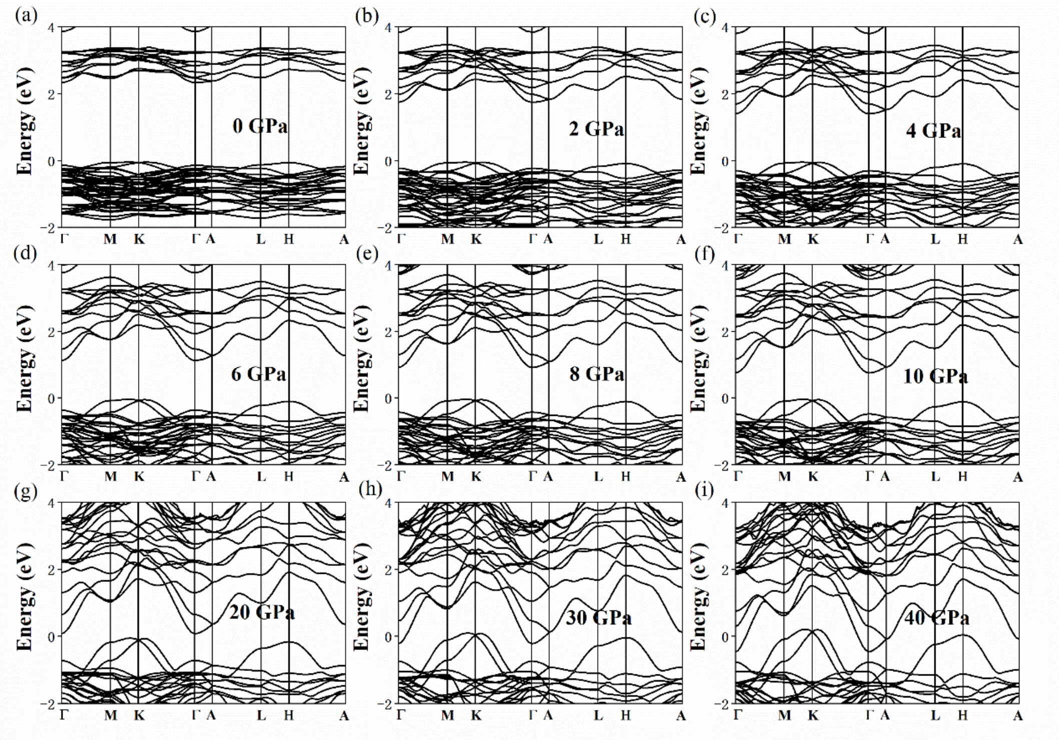


**Figure S1.** Calculated band structure of $Cs_3Bi_2I_9$ perovskite (a–i) under pressures from 0 to 40 GPa (d).

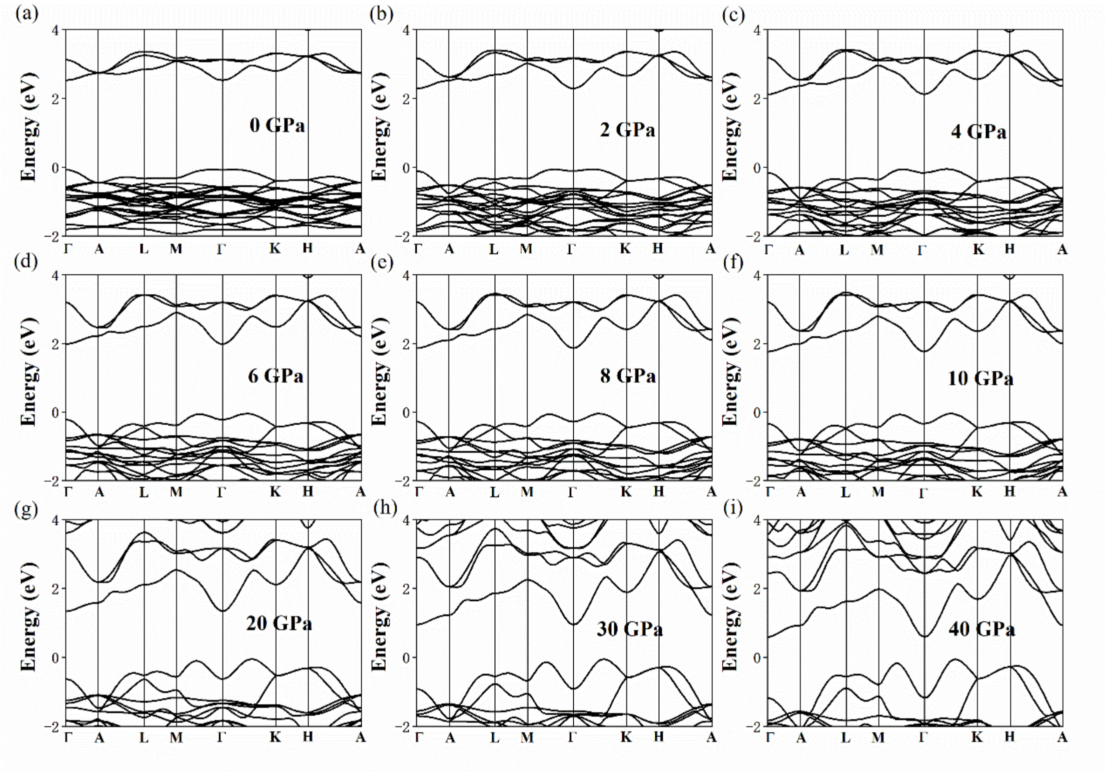


**Figure S2.** Calculated band structure of $Cs_3Bi_2Br_9$ perovskite (a–i) under pressures from 0 to 40 GPa (d).

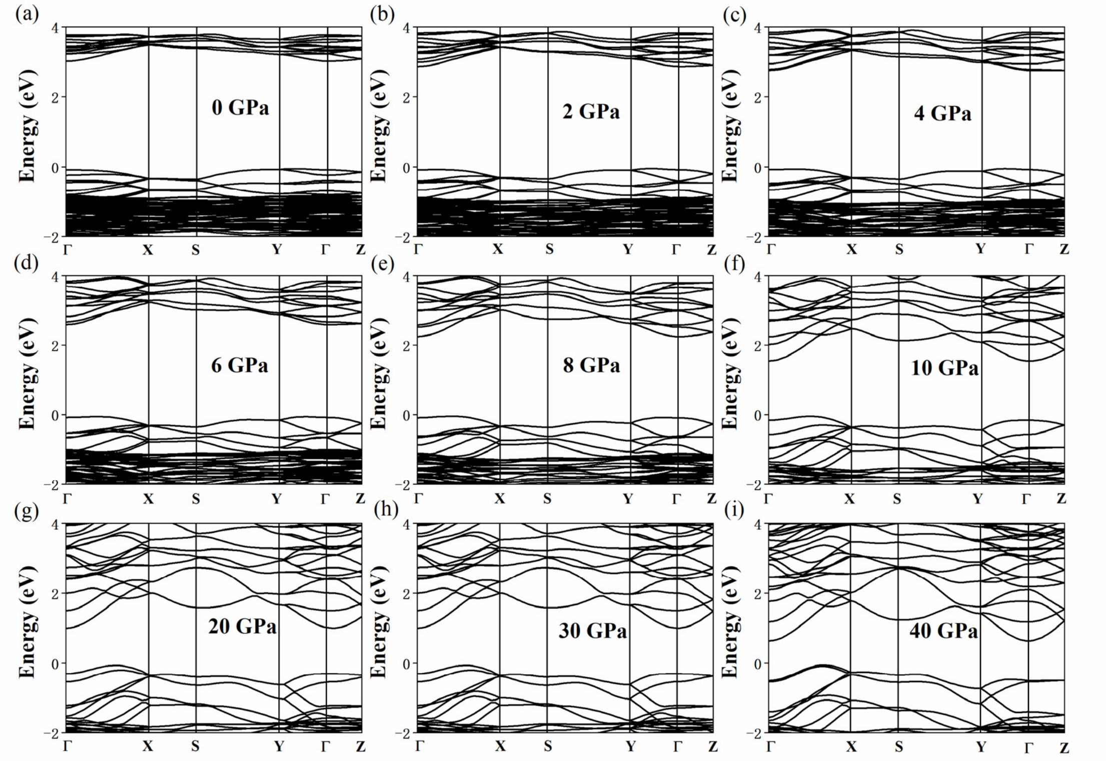


**Figure S3.** Calculated band structure of $Cs_3Bi_2Cl_9$ perovskite (a-i) under pressures from 0 to 40 GPa (d).

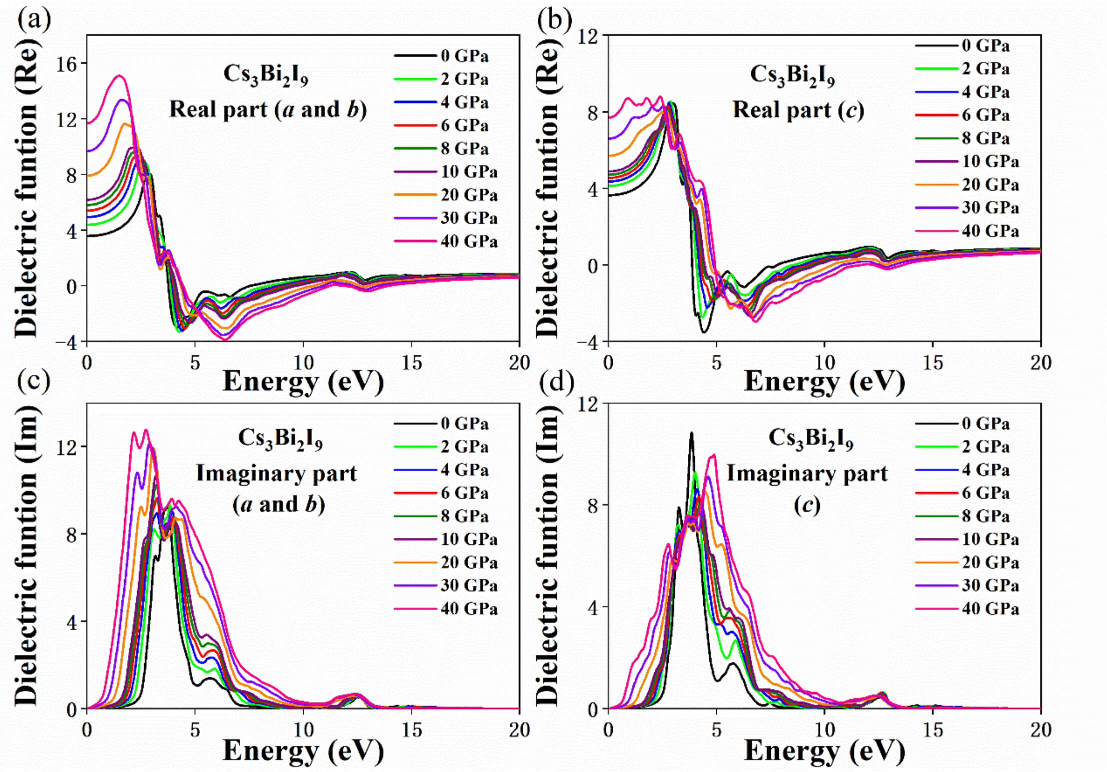


**Figure S4.** Real part of the dielectric function of the $Cs_3Bi_2I_9$ perovskite along the *a*- and *b*-axes (a), and the *c*- axis (b) as a function of pressure (from 0 to 40 GPa). Imaginary part of the dielectric function of the $Cs_3Bi_2I_9$ perovskite along the *a*- and *b*-axes (c), and along the *c*-axis) (d) as a function of pressure (from 0 to 40 GPa).

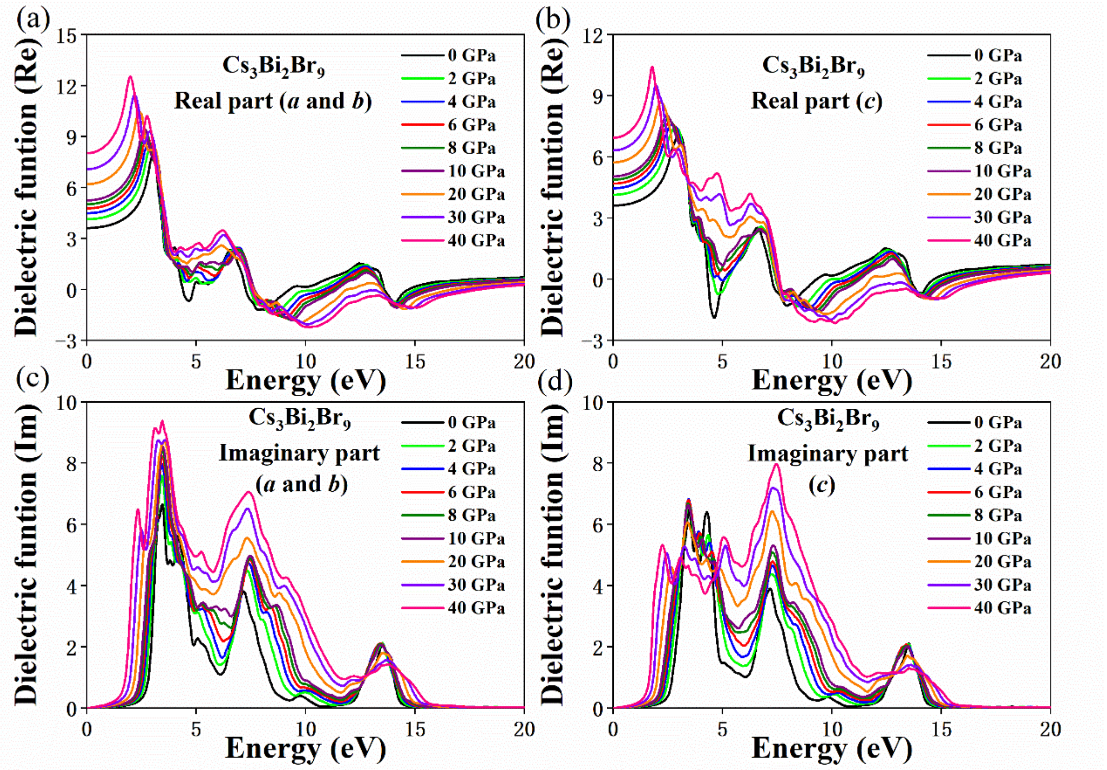


**Figure S5.** Real part of the dielectric function of the $Cs_3Bi_2Br_9$ perovskite along the *a*- and *b*-axes (a), and along the *c*-axis (b) as a function of pressure (from 0 to 40 GPa). Imaginary part of the dielectric function of the $Cs_3Bi_2Br_9$ perovskite along the *a*- and *b*-axes (c), and along the *c*-axis (d) as a function of pressure (from 0 to 40 GPa).

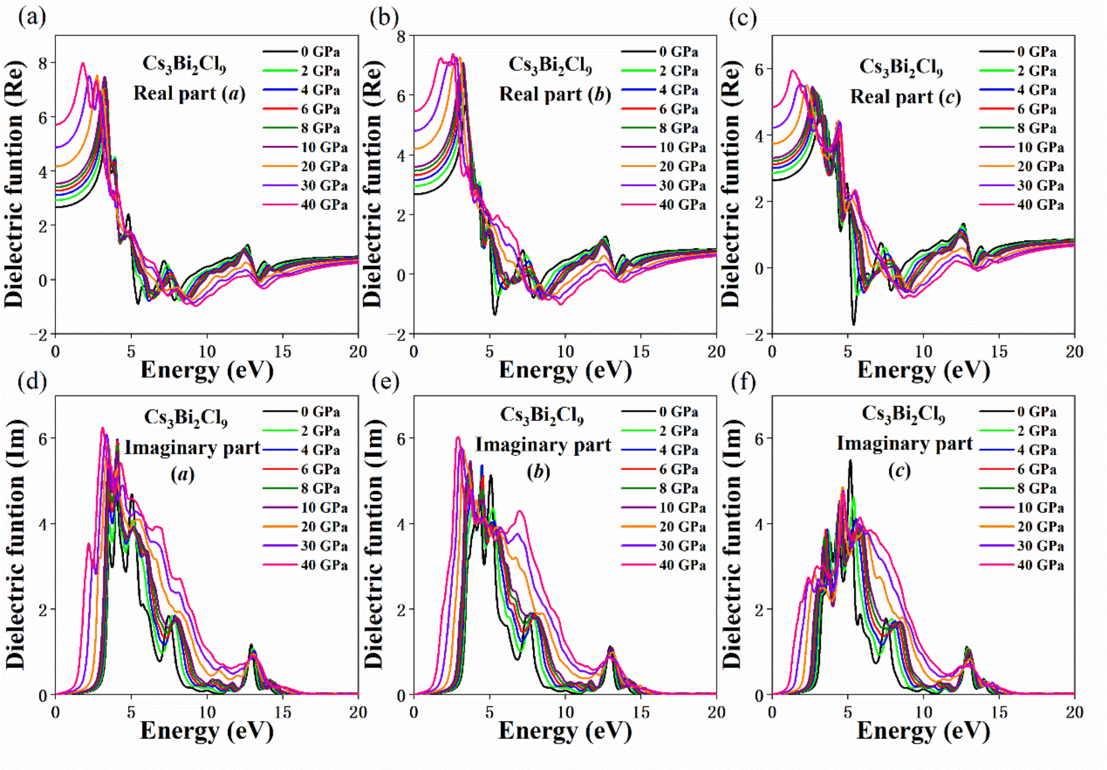


**Figure S6.** Real part of the dielectric function of the $Cs_3Bi_2Cl_9$ perovskite along the *a*-axis (a), along the *b*-axis (b), and along the *c*-axis (c) as a function of pressure (from 0 to 40 GPa). Imaginary part of the dielectric function of the $Cs_3Bi_2Cl_9$ perovskite along the *a*-axis (d), along the *b*-axis (e), and along the *c* axis (f) as a function of pressure (from 0 to 40 GPa).

## References

1. Zhang, L.; Liu, C.; Wang, L.; Liu, C.; Wang, K.; Zou, B. Pressure-Induced Emission Enhancement, Band Gap Narrowing and Metallization of Halide Perovskite $Cs_3Bi_2I_9$. Angew. Chem. Int. Ed. 2018, 57, 11213–11217.
2. Geng, T.; Wei, S.; Zhao, W.; Ma, Z.; Fu, R.; Xiao, G.; Zou, B. Insight into the structure–property relationship of two-dimensional lead-free halide perovskite $Cs_3Bi_2Br_9$ nanocrystals under pressure. Inorg. Chem. Front. 2021, 8, 1410.
